# The Influence of One Disease upon Another

**Published:** 1898-05-14

**Authors:** 


### THE INFLUENCE OF ONE DISEASE UPON ANOTHER.

The very interesting question raised by Dr. Harry Campbell, as to the beneficial influence of one disease upon another, brings up many problems in regard to which general practitioners ought to be able to throw much light.

**Metastasis.**—The first difficulty we meet with in considering the subject is that many of the curious cases where one form of disorder appears to recede as another comes to the front, may be only instances of metastasis. When, however, we ask ourselves what we mean by metastasis, and how we explain the sudden cessation of the first manifestation, we seem to see that, however true it is that both are but signs of the same morbid state, there is something in the development of the second which prevents the first from continuing its activity. It is impossible to conceive of disease as a something that hops about, so that we are forced to believe either that metastasis is a mere matter of the spreading of the disease to new parts, while in those first affected a local immunity is produced (a hypothesis which seems difficult to maintain in view of the occasional return of the malady to its old quarters), or that the development of the disease in the new focus in some way beneficially influences that which existed in the old.

**Trauma.**—Not only is it by no means rare for an accident to cure a disease, but it often seems to act beneficially upon the general health. The injury may, in some cases, do good by its local effect, as by the breaking down of old, painful, and disabling adhesions. In this there is nothing particularly surprising. Again, an accident may cause a patient ever afterwards to adopt a different mode of life, which, even if not a more healthy one, may, at any rate, protect him from injurious influences, as for example, when a fracture is followed by the cure of bronchitis or of rheumatism, as the result of the indoor life which it induces. Still another factor in the improved health which sometimes follows an accident is, no doubt, to be found in the fact that many active neurotic people are in a chronic condition of ill health in consequence of the constant wear and tear to which they expose themselves, and in which they delight. To these people an accident, which causes them to lie up and be well fed and carefully nursed for many weeks, not only gives a much-required rest, but it also sets them on a track of careful living which they follow for a long time afterwards, much to their advantage.

**Febrile Diseases.**—By far the most interesting cases, however, are those in which the occurrence of a febrile disease appears to exercise a beneficial effect upon a pre-existing malady. In regard to the explanation of these cases, the first thing to be remembered is that pyrexia is itself no doubt a defensive mechanism, and that the resistance to certain experimental infections is increased if before inoculation the temperature be raised by injuring the corpus striatum. Then it is not impossible that the development of the toxins of some diseases may, to some extent, interfere with the activity of other infections; while as a mere result of the altered metabolism which goes on during pyrexia, so great a difference in nutrition may be produced as to act beneficially upon disease. Diabetes, for example, may be held in abeyance, urethral and other discharges may dry up, and, as is well known, callus may be absorbed when fever intervenes.

In considering the action of fevers, however, upon more or less chronic preceding conditions, the nursing, the rest, and the feeding must again not be forgotten; and a case mentioned by Dr. Campbell, in which a chronic diarrhœa was found to have vanished after an attack of malignant typhoid, was probably one in which the occurrence of the fever led to a prolonged course of milk diet by which the diarrhœa was put right.

Still, outside and beyond such cases, Dr. Campbell gives several, and we have no doubt that the list could be very largely increased, in which the occurrence of an acute febrile disease appeared so to modify the nutrition as to be followed by the disappearance of morbid states which had been present for some time before, such, for example, as one striking case in which tuberculosis was much relieved by an attack of scarlet fever, and another in which the latter disease brought on menstruation in a case of anæmia and amenorrhœa, and was followed by permanent improvement in health.

The cases in which relief to mental symptoms followed the occurrence of acute febrile disease are very curious, and the effects of erysipelas on various morbid conditions, such as lupus and malignant growths, are too well known to require more than a mention.

The beneficial effect of vaccination again upon various conditions, such, for example, as eczema, is a matter which is strongly believed in by many.

The explanation of all these various cases is very difficult, but if we are to take it that febrile ailments do good by way of the different character of tissue metabolism which they induce, we can hardly avoid the conclusion that, so far as physiological chemistry is concerned, the difference between health and disease hinges upon a very small rearrangement of molecules—which is hopeful in regard to the therapeutics of the future.

